# Asymmetric Lipid Vesicles: Techniques, Applications, and Future Perspectives as an Innovative Drug Delivery System

**DOI:** 10.3390/ph16060777

**Published:** 2023-05-23

**Authors:** Denisse Gardea-Gutiérrez, Eduardo Núñez-García, Berenice E. Oseguera-Guerra, Manuel Román-Aguirre, Silvia L. Montes-Fonseca

**Affiliations:** 1 Tecnologico de Monterrey, School of Engineering and Sciences, Av. H. Colegio Militar 4700, Nombre de Dios, Chihuahua 31300, Chih, Mexico; 2 Centro de Investigación en Materiales Avanzados CIMAV, Av. Miguel de Cervantes 120, Complejo Industrial Chihuahua, Chihuahua 31136, Chih, Mexico; 3 Tecnologico de Monterrey, School of Medicine and Health Sciences, Av. H. Colegio Militar 4700, Nombre de Dios, Chihuahua 31300, Chih, Mexico

**Keywords:** drug delivery, asymmetric liposome, bioavailability, encapsulation efficiency

## Abstract

Novel lipid-based nanosystems have been of interest in improving conventional drug release methods. Liposomes are the most studied nanostructures, consisting of lipid bilayers ideal for drug delivery, thanks to their resemblance to the cell plasma membrane. Asymmetric liposomes are vesicles with different lipids in their inner and outer layers; because of this, they can be configured to be compatible with the therapeutic drug while achieving biocompatibility and stability. Throughout this review, topics such as the applications, advantages, and synthesis techniques of asymmetric liposomes will be discussed. Further, an in silico analysis by computational tools will be examined as a helpful tool for designing and understanding asymmetric liposome mechanisms in pharmaceutical applications. The dual-engineered design of asymmetric liposomes makes them an ideal alternative for transdermal drug delivery because of the improved protection of pharmaceuticals without lowering adsorption rates and system biocompatibility.

## 1. Introduction

Numerous drug delivery methods have been developed for therapeutic applications. Conventional drug delivery systems, such as tablets, capsules, lozenges, syrups, and ointments [1], transport therapeutic molecules without any type of control into the human body by oral consumption, injection, or topical administration. The disadvantages of these conventional systems include the need for biocompatibility, poor distribution, burst or disrupted release, low specificity [2,3], and sometimes side effects [4]. Drug delivery can be improved using nanocarrier-based systems to deliver therapeutic molecules in a controlled, directed, and efficient manner, particularly when combined with local administration [5].

There is a need for highly effective and less toxic alternatives to treat existing and emerging diseases. Historically, researchers have studied nanocarrier-based drug delivery systems, such as solid lipid nanoparticles, liposomes, polymeric micelles, metallic nanoparticles, nanoemulsions, and nanoliposomes, to improve their therapeutic effects against specific diseases, protecting the active molecule against degradation and reducing size effects [3]. In addition, these novel systems could be the key to promoting the development of individualized and molecular medicine strategies [5].

This review aims to show the advances in developing and using asymmetric liposomes as drug delivery systems. To reach this objective, we showcase the most relevant methodologies for developing asymmetric liposomes, their most outstanding applications, outlooks on the use of computational tools in the design of asymmetric liposomes, and their use as transdermal drug delivery systems.

## 2. Liposomes as Drug Delivery Systems

Nanocarriers such as liposomes can deliver therapeutic agents on target at required concentrations and, thanks to their large surface-to-volume ratio, they can transport large amounts of cargo with a smaller volume. These structures can even cross smaller networks of blood capillaries and provide efficient site-specific targeting, with prolonged and sustained releasing effects and fewer side effects, protecting entrapped molecules from chemical and enzymatic degradation [6,7,8,9].

Liposomes, one of the most studied nanocarriers in pharmaceutical areas, are versatile platforms for carrying and delivering drugs [10]. These nanoparticles are small artificial lipid vesicles with a spherical shape that consist of lipid bilayers from non-toxic phospholipids and, in some cases, cholesterol that surrounds aqueous units [11,12]. Liposome properties depend on the lipid composition, surface charge, size, and preparation methods; additionally, they require an extensive characterization of their final properties (vesicle size, surface charge, loading capacity, structural stability, and biocompatibility) before being used as biological systems [11,12].

**Figure 1:**
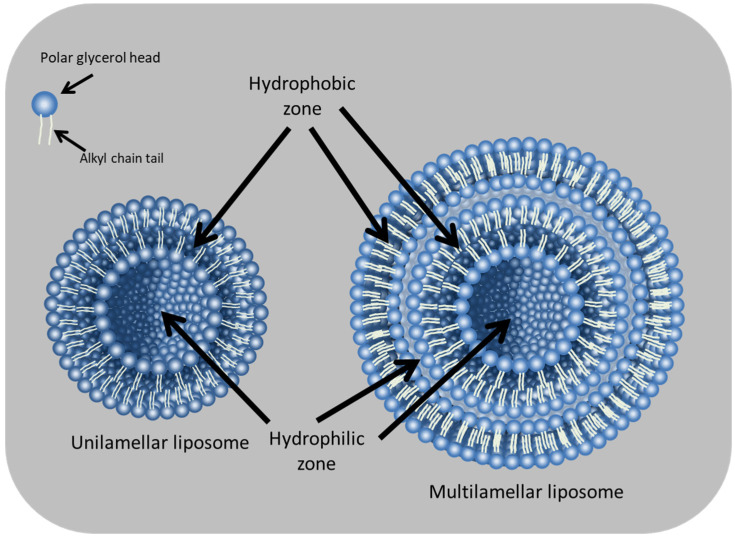
Structure of unilamellar and multilamellar liposomes.

**Figure 2:**
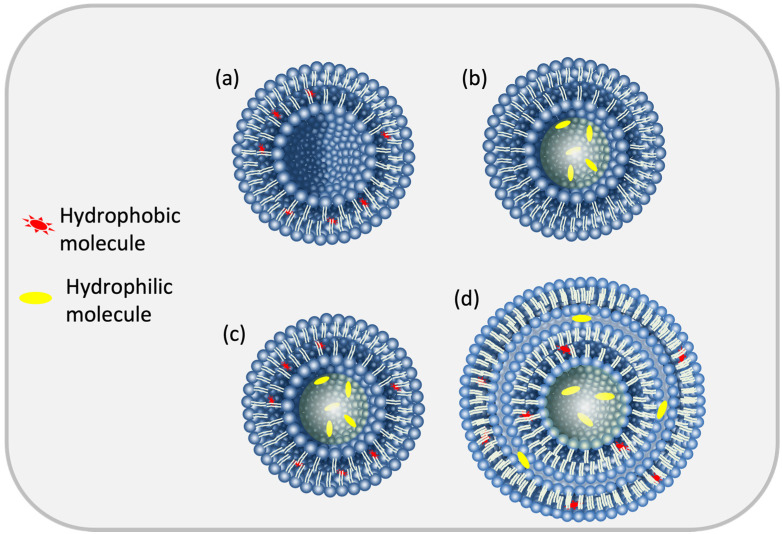
Versatile capacity of liposomes for different kind of molecules: (**a**) non-polar load; (**b**) polar load; (**c**) polar and non-polar loads; and (**d**) polar and non-polar loads into multilamellar liposomes.

Liposomes are ideal for drug delivery thanks to their capacity to entrap, shield, and release both hydrophobic and hydrophilic molecules (Figure 1 and Figure 2) and their resemblance to naturally occurring cell plasma membranes [11]. Compared with other nanoparticles, liposomes stand out as drug carriers by offering better biocompatibility and biodegradability, as well as lower toxicity [11,12].

**Figure 3:**
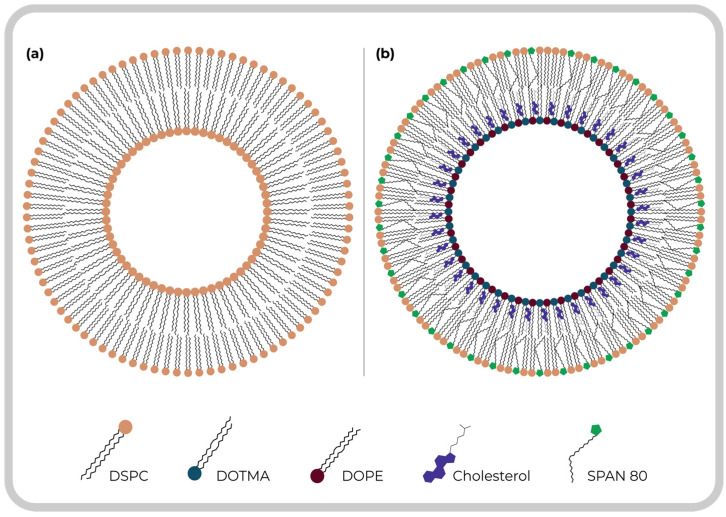
Representation of a comparison between a symmetric and asymmetric liposome. (**a**) Symmetric liposome composed only of DSPC; (**b**) Asymmetric liposome with different lipids in the inner (DOTMA, DOPE, and Cholesterol) and outer (DSPC and SPAN 80) layers.

In recent years, different types of liposomes have been developed; the most common are those considered symmetric liposomes (Figure 3a), which use the same lipid composition in the inner and outer parts of the lipid bilayer. Asymmetric liposomes (Figure 3b) are vesicles with lipid asymmetry, that is, the difference in lipid composition in the outer (exoplasmic) leaflet and the inner (cytoplasmic) leaflet of a membrane. Thus, asymmetric liposomes differ from conventional ones because they have different lipid compositions in the inner and outer layers [13,14,15], which seems to be more than the cell membrane lipid structure [16]. Having different lipids in each leaflet makes them relevant because of the possibility of optimizing the inner and outer layers independently to increase flexibility [12] and encapsulation efficiency [17,18]. Moreover, the leakage of drugs from the inner leaflet can be reduced, while different lipids can be used in the outer leaflet to enhance drug delivery and vesicle stability [17,18]. The flexible or deformable liposomes have special components such as edge activators (span 80, Tween 20, or ethanol) in the bilayers that decrease the interaction between lipids and favor the mobility of hydrophobic tails; this increases the potential for skin permeation, making it possible to have more compatible vesicular drug delivery systems used to protect many drugs for their biochemical and therapeutic purposes. Furthermore, the elasticity and membrane hydrophilicity promote avoiding vesicle aggregation due to osmotic stress, which usually limits conventional liposomes [18,19,20].

Although asymmetric liposomes are of great interest since they are a closer model to the cell membrane and a promising alternative as tailored drug carriers, preparing them is still a unique challenge. Several research groups have proposed methodologies for the preparation of asymmetric liposomes, such as successive phase interchanges of reverse emulsions [13], the use of cyclodextrins to exchange lipids between layers [21,22,23], and the use of microfluidics devices [24].

## 3. Synthesis of Asymmetric Liposomes

**Figure 4:**
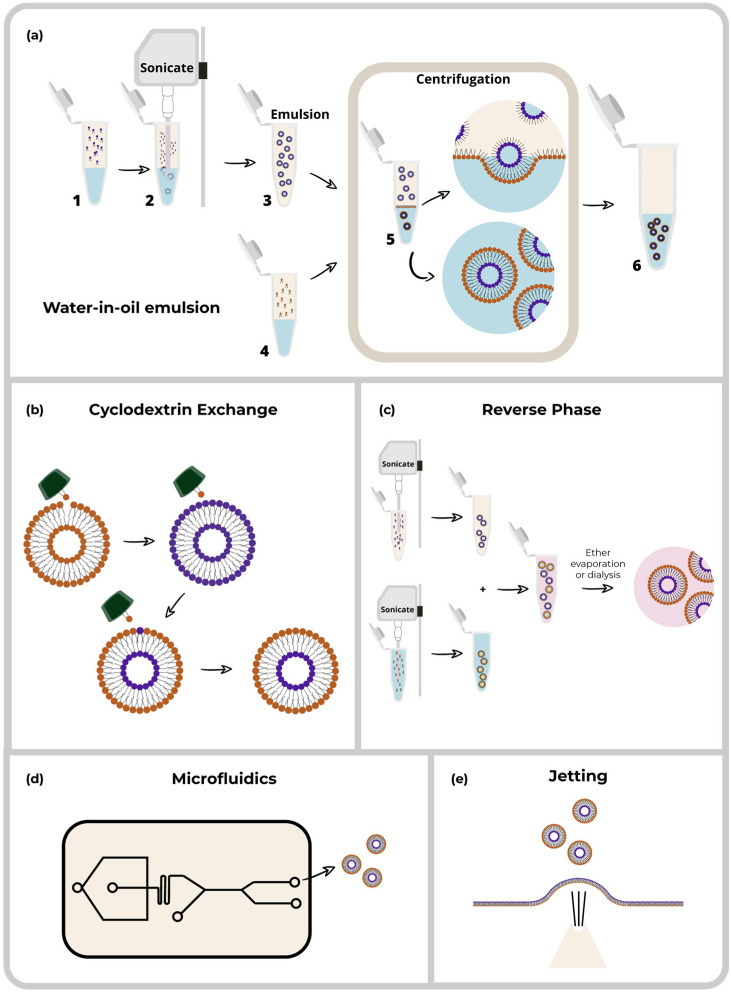
Methods of synthesis of asymmetric liposomes. (**a**) (1) Components of the inner layer are placed in the tube (2) Sonication is performed (3) Emulsion is created (4) Components of the outer layer are placed on the top of a buffer phase and allowed to be equilibrated until an oil-water interface is formed (5) The emulsion is placed on top of the formed interface (6) Centrifugation is performed, and asymmetric liposomes are collected from the aqueous phase.(**b**) Cyclodextrin exchange: the use of lipid-specific enzymes makes a lipid interchange between the outer leaflets of two different liposomes. (**c**) Reverse-phase evaporation: two inverted micelles are constructed through sonication, each mixture with different solvent composition but with ether as a shared component. Then they are mixed and the ether is evaporated or dialyzed to form a unilamellar vesicle. (**d**) Jetting: a high-pressure flow goes through a bilayer, forming vesicles. (**e**) Microfluidics: The use of a microchamber that allows for high-pressurized flow allows the formation of vesicles using methodologies based principally in *w*/*o* emulsions.

Over the years, researchers have developed multiple preparation methods for asymmetric liposomes. These methods continue to be improved and modified to obtain better encapsulation efficiency and stability, accomplishing more standardized processes. The most common methodologies described in this section are lipid exchanges through cyclodextrins, water-in-oil emulsions, reverse-phase exchange, jetting, and microfluidics, as summarized in Figure 4.

Many studies that apply these methodologies have successfully demonstrated the formation of asymmetric bilayers. Water-in-oil emulsions and enzymatic lipid exchanges are the most common and studied techniques, with water-in-oil emulsions standing out thanks to their capability to create asymmetric liposomes simply, making this technique attractive for the encapsulation of components such as nucleic acids [25]. On the other hand, the lipid exchange technique also has flexibility in comparison with other methods because it can exchange a wide variety of lipids; however, it requires more experience to be applied and is more time consuming. [26]. The most common methodologies are described below, as well as some of their most outstanding applications.

### 3.1. Water-in-Oil Emulsions

Asymmetric liposomes can be constructed using water-in-oil emulsions (*w*/*o*) through oil–water interfaces to form each leaflet of the bilayer independently [13]. It consists of three phases: the inverse emulsion, the intermediate, and the aqueous phases. The inverse or inverted emulsion of water molecules dispersed in oil is stabilized by the lipids intended for the inner leaflet; the amphiphilic nature of the lipids will collect around the water droplets with their hydrophobic tails pointed outward. Then the droplets of inverted micelles are passed through the intermediate phase of the same oil containing the lipids for the outer leaflet, which are at the oil–water interface between this and the next phase, with their hydrophobic tails pointing upward. After the droplets pass through the aqueous phase crossing the interface, a monolayer of the second lipid forms; the transfer process might occur spontaneously or using centrifugal force and/or sugar gradients [21].

This methodology has proven useful; for example, Whittenton et al. used this technique to generate immunoliposomes with DOTAP and DMPC in the inner leaflet and DMPC, POPC, NBD(7-nitro-2-1,3-benzoxadiazol-4-yl)amino]hexanoyl)-PC, and NBD-PS in the outer leaflet, encapsulating polynucleotides to evaluate the cellular uptake [27]. Additionally, de Matos et al. synthesized stable asymmetric liposomes of around 200 nm made from DOTAP and DSPC with a simple centrifugation-based *w*/*o* emulsion capable of entrapping pDNA [25].

### 3.2. Lipid Exchange through Cyclodextrins

Cyclodextrins (CDs) are cyclic oligosaccharides composed of six or more glucose units derived from the enzymatic degradation of starch. These have a hollow, truncated cone shape made up of the covalent link of the glucose units [28]. CDs have been an effective tool in transporting lipids because of their internal hydrophobic cavity, which binds to the lipid molecules’ acyl chains [29]. Due to this transport ability, CDs have been used to interchange lipids in the outer leaflet of artificial membranes or the plasma membranes of living cells with exogenous lipids [30]. This methodology was first implemented for asymmetric liposome construction in 2009 [31] and has since been used widely.

As observed in Figure 4b, the methodology depends on forming two vesicles: a donor and an acceptor. The donor vesicle is formulated as a multi-lamellar vesicle and added to a CD pool, and the acceptor is a unilamellar vesicle with a high-density internalized material. Then both vesicles interact thanks to the CDs that borrow lipids from the donor vesicles and exchange them with the outer layer of the acceptor vesicle [17].

The technique has been useful in preparing asymmetric molecules with different charges in their leaflets. Markones et al. presented a strategy to generate asymmetric liposomes precisely controlling the number of lipids exchanged for the outer leaflet; these liposomes had an outer leaflet composed of POPG and POPC with an inner leaflet of POPC [32]. Another study by Li and London used the CD exchange methodology to analyze the properties of large unilamellar vesicles’ LUVs with asymmetric charged leaflets for drug delivery. They found that having specific charges in each leaflet allowed better drug entrapment capacity within the vesicles [21]. This is because when using different lipids in each layer, the charges can be controlled depending on the drug to be encapsulated.

### 3.3. Reverse-Phase Evaporation

The original reverse-phase evaporation method tends to work similarly to that of *w*/*o* emulsions, in which bilayers are made through the passage between two phases. However, in this case, the bilayers are dissolved in the solvents to be evaporated, creating a thin film [33]; a simplified visualization of this process is shown in Figure 4c. The technique has not been widely used in the generation of asymmetric liposomes. Nevertheless, a study by Moktarieh et al. used a modified reverse-phase evaporation method, in which two inverted micelles with different lipid compositions were prepared separately in different solvents (DSPC, DOPE, and PEG-PE with an ether and citrate buffer for the outer micelle and DODAP and DOP with ether and HBS/ethanol in the inner micelle) and then mixed, followed by the evaporation and dialysis of the ether, allowing for the combination of both micelles. Symmetric liposomes of around 200 nm showed a 90% encapsulation efficacy of siRNA [34].

### 3.4. Microfluidics and Jetting

Microfluidics is not only one methodology; instead, several methodologies are applied through microfluidic systems, even though most microfluidics devices depend on common steps. Typically, a microfluidic device is composed of a stream of lipids (or a mixture of lipids) dissolved in an organic solvent flowing through a center channel meeting with streams of other media, usually aqueous, forming the vesicles represented in Figure 4d. In the case of asymmetric liposomes, the number of lipid streams is increased [35,36]. Microfluidics increases the probability of controlling the size of the vesicles and maintaining the homogeneity of the components of the layers [37].

Hu et al. used *w*/*o* emulsions combined with a microfluidics approach to generate giant asymmetric liposomes of multiple lipid compositions using DOPC, DPPC, cholesterol, biotin-DPPE, and biotin-DPPS [38]. Arriaga et al. reported a microfluidic approach for continuously producing asymmetric DOPC-DOPE-Biotinyl liposomes using a triple emulsion approach based on the *w*/*o* emulsions methodology [39]. Kamija et al. also used a combination of *w*/*o* emulsions with microfluidics to create asymmetric lipid giant vesicles with sizes of 3–20 μm and 100–200 μm made from DOPE, DOPS, and DOPC to investigate the dynamic responses of lipid molecules in the vesicle membrane [40].

The jetting technique is a variant of microfluidics that uses a pulsed jet flow against two parallel planar asymmetric lipid bilayers, as observed in Figure 4e. Therefore, to generate asymmetric liposomes, an asymmetric planar bilayer is necessary before applying the jet flow. The bilayer is created by two lipid streams mixed in an intermediate point with a solvent phase in which the asymmetry is conserved [41]. This method has been used to produce asymmetric liposomes with a size of approximately 23.6 μm made of DOPC, DOPS, DOPE, rhodamine-DOPE, and NBD-DOPE. Subsequently modifications to the methodology in the jet flow duration for an extra 6 ms with an increased pressure of 0.5 MPa instead of 0.3 MPa allowed for the generation of smaller vesicles made from DOPE, rhodamine-DOPE, biotin-DOPE, and PEG(2000)-DSPE with a size of approximately 200 nm [42]. These techniques require fabricating customized devices to synthesize liposomes according to the necessities and type of the desired product.

## 4. Asymmetric Vesicles: Advantages and Applications

Liposomes have been widely studied for drug release applications, for which they are known to have the desired effect by releasing specific concentrations on site. In drug delivery, when a drug is supplied directly to the bloodstream, problems such as short circulation times, drug breakdown, and clearance are lessened [21]. Liposomes are an alternative to avoid these problems because they can trap the drug, control the dosage need, and have an effective drug concentration to target the desired cells [43].

In recent years, the applications of asymmetric liposomes have been gaining importance due to their benefits. Synthetic asymmetric bilayers mimic biological functions better than their symmetric counterparts because naturally occurring bilayers have an asymmetric behavior. Because of this, constructing these types of vesicles represents a step forward in understanding cell membranes and would allow for better delivery systems with cells as a target [32,42,44]. Asymmetric liposomes are also a helpful model system for the in vitro analysis of lipid–lipid and protein–lipid interactions and improve our understanding of cellular processes [26]; innovative and novel models must be developed.

Asymmetric liposomes have properties that can optimize drug delivery due to their capacity to have different lipids in their layers and to control characteristics such as the charge. For example, it has been exhibited that a high charge density in the inner leaflet helps for an efficient condensation of biopharmaceuticals, such as nucleic acids, and simultaneously, a neutral or negative outer layer is more suitable for biocompatibility [45]. Some advantages of asymmetric lipid vesicles in different research applications are shown in Table 1.

### 4.1. Asymmetric Liposomes for Drug Delivery

Liposomes are representative of the development of new and better delivery systems regarding the enhancement of encapsulation, release, and efficiency. One of the objectives of this section is to describe the advantages and applications of asymmetric liposomes in drug delivery systems; a summary of studies related to this application is displayed in Table 2. London et al. developed asymmetric liposomes containing cationic or anionic outer leaflets and inner leaflets that had either the opposite charge or were uncharged, and the diameter of the asymmetric liposomes was around 120 nm. They found that anionic lipids in the inner leaflet maximized the amount and stability of doxorubicin entrapment within the vesicles, suggesting that it is possible to choose inner leaflet lipids to maximize the liposomal loading of charged drugs, and the outer leaflet should favor the bioavailability and biodistribution of the vesicles [21].

Moreover, asymmetric liposomes can be helpful as alternative therapeutic strategies, as the research by Greco et al. shows, in which asymmetric liposomes were designed to act as apoptotic bodies that killed Mycobacterium tuberculosis bacteria without antibiotics to alleviate the incidence of antibiotic resistance in tuberculosis treatment. This therapy was successfully applied to mice in an inhalable route [17,46]. They used the inverted emulsion technique taken from Pautot et al. with phosphatidylserine in the outer membrane to resemble apoptotic bodies as well as phosphatidic acid in the inner layer to enhance the innate antimycobacterial activity in phagocytes while limiting the inflammatory response. They concluded that the possibility of distributing lipids in the liposome membrane asymmetrically is additionally of value in liposome-based therapeutic strategies because of the cargo of bioactive lipids, which can be used as unique immunomodulators, and can be preferentially delivered to specific target cells [46,47].

Jing et al. designed asymmetric lipid membranes in which the asymmetry was generated through the selective PEGylation of cationic lipids in the outer membrane leaflet, so an asymmetry between two membrane leaflets of liposomes was created while the charged surface function at the outer liposome surface of the symmetric liposomes was deactivated. This study mentions the importance of designing improved anticancer drugs and of using drug carriers in combination therapies [48].

Asymmetric-type liposomes continue to be studied for their application in the release of different drugs, so it is expected that in the future, the control over the layers of these nanosystems, as well as the benefits that these kinds of liposomes have, will lead to their application in drugs that require a more sustained release as well as better doses and routes of administration.

### 4.2. Asymmetric Liposomes for Nucleic Acid Delivery

In molecular medicine, nucleic acid therapeutics is one of the most significant advances encouraging the development of new technologies [25,45]. Using nucleic acids in therapy has disadvantages, including that molecules cannot enter or transfect the cell by themselves, mainly because of the negative charge of nucleic acids, which makes it difficult for them to pass through cell membranes. All nucleic acids exceed the size of conventional small drugs, and they are easily degraded by the nucleases present in physiological fluids, leading to limited biological stability [25,45]. For this reason, when delivering genetic material to the human body, it needs a carrier that protects and transports the nucleic acids safely [17,51].

Asymmetric lipid particles have proven to be an efficient tool for studying nucleic acid delivery due to their ability to control the inner and outer layer charge. It has been shown that a high positive charge density in the inner part is an advantage for the efficient encapsulation of nucleic acids. At the same time, a neutral or negative outer layer is an advantage for biocompatibility [29]. Cationic lipids are studied in the design of asymmetric liposomes, especially for the inner layer, where the cationic lipids form complexes with the nucleic acids, which are anionic [52]. Good encapsulation efficiencies and stability of the encapsulated nucleic acids in this type of vesicles have been observed (Table 2), but the encapsulation efficiency also depends on the synthesis technique.

De Matos et al. made improvements in and contributions to nucleic acid encapsulation using the inverse emulsion technique. They used positive phospholipids (DPPC/DOTAP) in the inner leaflet and (DPPC/DSPE-PEG2000) in the outer. The final liposomes had sizes below 200 nm and a bilayer asymmetry of 70%, making this an attractive methodology for encapsulating nucleic acid therapeutics because of the final size of the liposomes, genetic material integrity, and the successful production of the asymmetric bilayers. This study was the first report in which centrifugation technology was employed for the production of nanosized liposomes for pDNA encapsulation using the inverse emulsion technique [25]

Mokhtarieh et al. developed a method for making asymmetric liposomes with a high siRNA encapsulation efficiency (90%) and negatively charged surface that precludes nonspecific liposome uptake into cells. The inner layer was composed of ionizable cationic DODAP and DOPE, which entrap siRNA, and the outer layer was composed of DSPC, DOPE, PEG-PE, and cholesterol. Moreover, these vesicles protect siRNA from ribonuclease A degradation, and the conjugation of the outer layer with different molecules induces mediated uptake into specific cells. These findings suggest that asymmetric lipid nanoparticles could be valuable cargo for delivering target-specific siRNA [34].

A suitable vehicle with a high capacity for nucleic acid loading and one that allows for an effective and timely release in the nucleus or cytoplasm of the cells is typically required for successful targeted delivery and efficient gene transfection outcomes. Inefficient delivery vectors can compromise therapeutic advances and, eventually, the potential of gene therapy [53]. Because of this, the development and study of asymmetric liposomes represent an opportunity for advances related to therapies in molecular medicine and pharmaceutical areas.

### 4.3. Cell Models

To study the biophysical characteristics of biological cell membranes, in vitro simplified systems are necessary. While artificial lipid bilayers are useful to model natural membranes, they are generally symmetric and lack at least some of the critical structural characteristics of natural cell membranes. Since cellular membranes have different lipid domains in different parts of the membrane that are required to control different cellular activities that are essential for differentiation, proliferation, protein interactions, and cell-to-cell communication [48,49,54], symmetric bilayers lack the capacity to emulate these behaviors, thus limiting the information obtained from working with this kind of model. Therefore, asymmetric systems could improve our understanding of cellular processes due to their resemblance with the asymmetric behavior of eukaryotic or bacterial cellular membranes [49,55,56].

Lipid asymmetry has several roles in biological processes; for example, the display of phosphatidylserine in the outer leaflet of cell membranes signals the consumption of apoptotic cells by phagocytes [57]. Moreover, lipid charge asymmetry allows for determining the orientation of proteins in the membrane [58]. Another aspect likely affected by asymmetry is the physical lipid state (i.e., liquid-ordered and disordered states), which affects cellular functions such as amyloid formation, protein, lipid sorting, cell signal transduction, and pathogen invasion [15].

A study that pushed forward synthetic models resembling naturally occurring membranes was presented by Lin et al., which concluded that the cholesterol-containing asymmetric liposomes made from SM/POPC (inner leaflet) and POPE/POPS (outer leaflet) closely resemble mammalian plasma membranes because their vesicles emulate essential features, such as lipid composition and asymmetry [59].

Kamiya et al. demonstrated that the formation of asymmetric lipid vesicles with the inner leaflet of DOPS/DOPC (with a 1:1 molar ratio) and outer leaflet of DOPC emulated lipid flip-flop corresponding to the apoptotic cells’ behavior and showed the promotion of the flop dynamics influenced by an antibiotic peptide. Additionally, these vesicles achieved at least seven days of long-term storage stability through microfluidics-based preparation methods [40].

Doktorova et al. measured lipid flip-flop using time-resolved small-angle neutron scattering (SANS) to study the asymmetric bilayers’ stability. They concluded that asymmetric liposomes are better as biological mimetics than their symmetric counterparts due to the alterations in lipid lateral diffusion, packing density, phase behavior, and the conformation, partitioning, and topology of transmembrane proteins. They produced asymmetric large unilamellar vesicles (aLUVs) through a lipid exchange methodology and experimented with different lipid combinations [26].

Asymmetric liposomes allow for the analysis of structural interactions resembling the ones naturally occurring in plasma membranes; therefore, the study and innovation of more realistic models are critical to developing strategies to understand how drugs could interact in vivo with cellular membranes.

## 5. Computational Tools for the Design of Asymmetric Liposomes

Many disadvantages in the formulation and characterization of asymmetric liposomes have been reported [20]. Some parameters, such as stability, the degree of asymmetry, and asymmetric vesicle stability, are not evaluated because the existing analytical techniques are not specific for measuring these parameters, and some are complicated or require expensive equipment [22,60]. Fluorescence, for example, can be used to measure asymmetry in the liposomes, but fluorophores attached to lipids can change liposomal properties. Additionally, high amounts of quencher might be required for the fluorescence analysis which might increase osmolarities outside of the liposomes affecting liposomal characteristics [49].The use of computational tools such as Molecular Dynamics (MD) simulations applied to lipid bilayers’ membranes allows for the exploration of various thermodynamic and kinetic processes and is a valuable tool in addressing issues that are difficult to explore in laboratory experiments [61,62].

MD simulations provide three-dimensional, real-time structural and dynamic information about lipidic systems which would hardly be accessible by any experimental method [63]. The Martini force field is a coarse-grained (CG) model particularly intended to study lipid membrane properties. This tool allows for the analysis of the behavior of large lipid aggregates at spatial and temporal scales to provide microscopic and dynamic information unavailable to experimental data [64,65]. As a CG model, it involves the description of microscopic particles at a mesoscopic level by grouping atoms into clusters to reduce the number of calculated interactions while allowing us to understand the underlying dynamics of the lipidic system [62,66]. This model has been used to research vesicle formation [67], the roles of lipid compositions on liposomal formulations, flip-flop motions in lipid membranes [68], realistic membrane models [69,70,71,72], and the behaviors of lipidic structures [62,73,74]. MD simulations can be a valuable tool to predict the lipid structure and stability of a mixture of lipids that make up an asymmetric bilayer. Our laboratory is currently working on in silico studies of asymmetric liposomes to predict the best lipid mixture that would result in more stable vesicles and more favorable assembly thermodynamics.

## 6. Future Perspectives in the Use of Asymmetric Liposomes as Transdermal Delivery System

The direct delivery of drugs into the body is challenging due to the poor distribution of therapeutic agents, off-target side effects, and a short circulation time, given the breakdown and clearance of the drug [75]. For example, in most cases of drug administration through oral delivery, therapeutic treatment is not achieved due to hepatic first-pass metabolism, adverse side effects, and the rejection of invasive treatments [76,77]. Moreover, drugs’ solubility issues in the intestinal fluid and permeability through the intestinal membrane act as rate-limiting steps in drug absorption, causing low bioavailability [78].

Transdermal drug delivery represents an attractive and innovative alternative; with this route, the possibility of achieving a systemic delivery of drugs is promising [79]. Fruthermore, this scenario demands a particular focus on developing strategies for minimizing toxic adverse effects related to most pharmacologically active agents. Exploring new alternatives, such as the design of liposomal nanosystems, is one of the most promising options for achieving a successful strategy for the better release of active agents.

Liposomal nanosystems facilitate drug transport through the skin and are a promising alternative to oral or intramuscular systems [80]. Liposomes can be created from cholesterol and natural, non-toxic phospholipids compatible with the skin’s surface. The design and development of flexible liposomes composed of complex mixtures of surfactants and edge activators that improve permeation in the skin due to having a characteristic flexible membrane are considered a promising tool for the release of substances with biological activity [79].

Notable advantages of asymmetrical liposomes allow a better design control because of the selection of the inner layer components, promoting the stability and functionality of the internalized therapeutic compound and maximizing the loading capacity. Additionally, depending on the delivery route, the outer leaflet can be engineered to improve biocompatibility, increase the absorption rate, and accurately deliver to specific cells. This advantage leads to more delivery routes, uses, and stability in handling therapeutic compounds and drugs. It is also important to mention that the synthesis of asymmetric liposomes is still in development, and the various techniques can be helpful depending on the type of encapsulated substance. Asymmetric type liposomes have potential in the study of transdermal delivery, so their study and application are a relevant research area regarding the design of a liposome that is flexible and can be applied transdermally, making it possible to obtain all the benefits that this route offers.

As the nanotechnology field evolves, researchers can achieve success in the design of delivery systems by taking advantage of other administration routes, such as transdermal or inhalable ones, which represent an excellent opportunity to switch from invasive drug delivery systems, for example, vaccines.

Therefore, there is a need to develop more efficient, biocompatible, and safer treatments in molecular medicine for patient compliance. Nanotechnology has the potential to build and design new drug delivery systems, such as asymmetric liposomes, which are promising tools for encapsulating a wide range of molecules that require improved bioavailability.

## 7. Conclusions

Designing new and better transport vehicles for therapeutic molecules is a recent, highly relevant topic. Asymmetric liposomes have shown to be a successful alternative in encapsulating biological molecules; also, their ability to mimic biological membranes makes them attractive in studying biological-membrane-related processes.

One of the advantages of asymmetric liposomes is their duality of chemical properties inside and outside the vesicle. This characteristic allows us to load different molecules and keep them stable with better biological activity. In the same way, the design of the outer leaflet is an advantage in directing and transporting the load efficiently to a biological target. For this reason, we find this system useful as a transdermal drug delivery system, in which we can design flexible and biocompatible structures for their efficient absorption through the stratum corneum of the skin.

However, a disadvantage of this system is the lack of standardization in terms of its synthesis and characterization methods. Until now, some synthesis methods have not been reproducible, and the distribution of lipids between the layer of the bilayer is difficult to characterize. For this reason, the use of computational tools supports the understanding of the behavior of lipids within the asymmetric system, as well as in predicting their stability, compatibility with the charge and the solvent, and even their pharmacokinetic behavior.

## Figures and Tables

**Table 1:** Advantages of asymmetric lipid vesicles in different research applications.

General Drug Delivery	Nucleic Acids Delivery	Cell Models
Capacity to enhance the properties of the inner and outer leaflets independently to optimize the composition depending on the drug to encapsulate.	Protects nucleic acids from degradation.	A helpful model system for the in vitro analysis of lipid–lipid and protein–lipid interactions.
Different lipids can be used in the outer leaflet to enhance drug delivery and vesicle stability.	A neutral or negative outer layer is an advantage for biocompatibility.	Crucial in developing strategies to understand how drugs could interact with cellular membranes.
Asymmetric liposomes can be engineered to target specific cell types.	Inner-layer engineering allows for better encapsulation capacity.	Better at biological mimetics than their symmetric counterparts.

**Table 2:** Summary of asymmetric liposome studies with therapeutic applications. In some studies, the data is not presented, and it is indicated by a hyphen (-).

Synthesis Technique	Inner Leaflet	Outer Leaflet	Molecule	Size	Stability	Encapsulation Efficiency	Ref.
Modified reverse phase evaporation method	DODAP/DOPE	DSPC/DOPE/PEG-PE/cholesterol	siRNA	200 nm	siRNA encapsulated was protected from enzyme degradation for up to 24 h	90%	Mokhtarieh et al., 2012 [34]
Inverse emulsions	DMPC/DOTAP	DMPC/POPC/NBD-PC	siRNA	44, 188, and 489 nm	Modes remain relatively constant over a 160 h period	-	Whittenton et al., 2013 [27]
Modified reverse phase evaporation method	DODAP/DOPE	DSPC/DOPE/mPEG-PE/miPEG-PE/cholesterol	Calcein and indocyanine green (ICG).	-	-	90%	Lee., 2015 [49]
Cyclodextrin-catalyzed lipid exchange method	DOPE:POPS, phosphatidylserine (PS)-	bSM, DOPE	-	-	Up to 48 h	-	Petazzi et al., 2015 [50]
Inverse emulsions	DPPC/DOTAP	DPPC/DSPE-PEG2000	plasmid DNA	200 nm	-	10–15%.	De Matos et al., 2019 [21]
Cyclodextrin-catalyzed lipid exchange method	POPC, POePC, DOTAP, POPS, POPG, POPA	POePC, POPC, DOTAP, POPS, DOTAP, POPG	Doxorubicin	120 nm	Not changing significantly in the first 48 h	Up to 13 µM Dox/mM Lipid	London et al., 2020 [25]

## Data Availability

Data sharing not applicable.
